# The rat pancreatic body tail as a source of a novel extracellular matrix scaffold for endocrine pancreas bioengineering

**DOI:** 10.1186/s13036-018-0096-5

**Published:** 2018-04-27

**Authors:** Huajun Yu, Yunzhi Chen, Hongru Kong, Qikuan He, Hongwei Sun, Pravin Avinash Bhugul, Qiyu Zhang, Bicheng Chen, Mengtao Zhou

**Affiliations:** 1 Department of Surgery, The First Affiliated Hospital of Wenzhou Medical University, Wenzhou, 325035 China; 2 Key Laboratory of Diagnosis and Treatment of Severe Hepato-Pancreatic Diseases of Zhejiang Province, Zhejiang Provincial Top Key Discipline in Surgery, Wenzhou, China

**Keywords:** Decellularization, Extracellular matrix scaffold, Recellularization, Organ bioengineering, Transplantation

## Abstract

**Background:**

Regenerative medicine and tissue engineering are promising approaches for organ transplantation. Extracellular matrix (ECM) based scaffolds obtained through the decellularization of natural organs have become the preferred platform for organ bioengineering. In the field of pancreas bioengineering, acellular scaffolds from different animals approximate the biochemical, spatial and vascular relationships of the native extracellular matrix and have been proven to be a good platform for recellularization and in vitro culture. However, artificial endocrine pancreases based on these whole pancreatic scaffolds have a critical flaw, specifically their difficult in vivo transplantation, and connecting their vessels to the recipient is a major limitation in the development of pancreatic tissue engineering. In this study, we focus on preparing a novel acellular extracellular matrix scaffold derived from the rat pancreatic body tail (pan-body-tail ECM scaffold).

**Results:**

Several analyses confirmed that our protocol effectively removes cellular material while preserving ECM proteins and the native vascular tree. DNA quantification demonstrated an obvious reduction of DNA compared with that of the natural organ (from 931.9 ± 267.8 to 11.7 ± 3.6 ng/mg, *P* < 0.001); the retention of the sGAG in the decellularized pancreas (0.878 ± 0.37) showed no significant difference from the natural pancreas (0.819 ± 0.1) (*P* > 0.05). After transplanted with the recellularized pancreas, fasting glucose levels declined to 9.08 ± 2.4 mmol/l within 2 h of the operation, and 8 h later, they had decreased to 4.7 ± 1.8 mmol/l (*P* < 0.05).

**Conclusions:**

The current study describes a novel pancreatic ECM scaffold prepared from the rat pancreatic body tail via perfusion through the left gastric artery. We further showed the pioneering possibility of in vivo circulation-connected transplantation of a recellularized pancreas based on this novel scaffold. By providing such a promising pancreatic ECM scaffold, the present study might represent a key improvement and have a positive impact on endocrine pancreas bioengineering.

**Electronic supplementary material:**

The online version of this article (10.1186/s13036-018-0096-5) contains supplementary material, which is available to authorized users.

## Background

Despite numerous investigations searching for appropriate treatment options, diabetes mellitus remains one of the most serious global chronic diseases [1, 2]. The current treatment for type I diabetes is mainly dependent on long-term insulin injection, but this approach does not represent a cure and can potentially lead to long-term complications, including kidney or heart damage [3–5]. β-Cell replacement therapy through islet or pancreas transplantation is the only reliable method for maintaining a stable glycemic state based on physiological insulin secretion. However, the clinical application of islet and pancreas transplantation has been hindered by factors such as a shortage of donors and chronic toxicity due to lifelong immunosuppression [6].

Developments in tissue engineering have prompted advances in the replacement of tissues or organs [7–9]. As a tissue engineering approach, extracellular matrix (ECM) scaffolds obtained via decellularization perfusion preserve the natural tissue morphology and biological characteristics, including an intact three-dimensional anatomical architecture, the natural arrangement of ECM components, and the vascular network, which might provide an attractive platform for the proliferation, differentiation and survival of cells [2, 10, 11]. In the field of pancreas bioengineering, seeding islet cells on scaffolding material appears to offer the quickest route to clinical application for diabetes therapy [12]. Many investigations have focused on successfully constructing natural pancreatic ECM scaffolds, which are characterized by a unique ECM composition and constitute an appropriate microenvironment; furthermore, several studies have demonstrated that extracellular matrix proteins and structures can play fundamental roles in maintaining the survival, proliferation and function of seeded islet cells [10, 13–15].

A future breakthrough in diabetes therapy is likely: the in vivo transplantation of a functional bioengineered endocrine pancreas constructed by seeding specific cells induced from stem cells onto scaffolds or a 3D-printed organ. However, there is currently almost no information on the in vivo application of transplanted recellularized pancreatic scaffolds or artificial endocrine pancreases based on an acellular scaffold. It is well established that a suitable scaffold for tissue engineering should meet the following conditions [16–18]: (1) the scaffolds should be easy to explant and be decellularized; (2) the scaffolds should retain natural structural relationships, ECM components and biochemical properties, providing the cues necessary for cellular adhesion and proliferation; (3) the scaffolds should be amenable to recellularization via perfusion; and (4) the scaffolds should also conserve the arterial inlet, venous outlet, and original circulatory pathway, enabling in vivo transplantation such that the circulation is connected to ensure an adequate supply of blood oxygen and nutrients. It appears that the existing pancreatic acellular scaffolds are not suitable for the study of in vivo transplantation due to the methodological limitations inherent in the decellularization perfusion of the complete pancreatic scaffold via the portal vein or bile duct. The anatomy of the pancreas includes multiple arterial blood supplies. The head of the pancreas is tightly wrapped by the duodenum, with extensive communicating branches between these organs [19], such that complete pancreatic acellular scaffolds have two major shortcomings: (1) the arterial inlet of these scaffolds for transplantation is not definite and clear and (2) the pancreatic heads of these scaffolds lack vascular closure and leak easily. We predict that even if a bio-artificial endocrine pancreas is successfully established based on a pancreatic acellular scaffold, failure to implant the pancreas in vivo with vascular connections between the donor and recipient or graft bleeding soon after transplantation will make the bio-artificial endocrine pancreas useless for diabetes therapy. Thus, we propose the preparation of a novel pancreatic acellular scaffold suitable for endocrine pancreas bioengineering.

In this study, we aimed to introduce a new decellularization strategy to prepare a partial pancreatic ECM scaffold derived from the rat pancreatic body tail (pan-body-tail ECM scaffolds) and to determine whether this type of pancreatic scaffold could serve as a suitable platform for a bioengineered endocrine pancreas, with an emphasis on in vivo transplantation applications. To this end, we demonstrated that the rat pancreatic body tail could be easily explanted and completely decellularized, with retention of its natural ECM composition, three-dimensional (3D) spatial structure and vascular channels. The arterial inlet and venous outlet of the pan-body-tail ECM scaffolds for transplantation were both subsequently defined and evaluated, and the resistance to circulatory leakage was determined. In addition, recellularization of the pan-body-tail ECM scaffolds with INS-1E cells and endothelial cells was successfully completed before in vivo transplantation. Furthermore, in vivo analyses were performed to evaluate the effects of transplantation on the modulation of blood glucose in diabetic rats and the histopathology and blood circulation of recellularized scaffolds after implantation.

## Methods

### Harvesting of the rat pancreatic body tail

Adult male Sprague Dawley (SD) rats weighing approximately 250 g were obtained from the animal center at Wenzhou Medical University. All animal work was performed according to the Animal Welfare Act and approved by the Animal Ethics Committee at Wenzhou Medical University, which conforms to the Guide for the Care and Use of Laboratory Animals published by the US National Institutes of Health (NIH publication no. 85–23, revised in 1996). The rats were anesthetized using pentobarbital sodium through intraperitoneal injection, and sterile conditions were maintained during the operation. A laparotomy was performed, and the celiac artery and its branches, including the common hepatic artery, left gastric artery and splenic artery, were exposed. The *Wistar Rat Anatomical Atlas* and our observations of the anatomy of the pancreas (Additional file 1) showed that the blood supply to the pancreatic head originated from the gastroduodenal artery, whereas that of the pancreatic body tail came from a branch of the splenic artery. A No. 10 polyethylene (PE) tube (China Ningbo Anlai Software & Equipment Co., Ltd.) was inserted approximately 1.0 cm into the left gastric artery and sutured in place. The celiac artery and the common hepatic artery were carefully ligated, and the splenic artery and its branches to the spleen were also thoroughly ligated to prevent leakage. Differences between the head and body tail of the pancreas were obvious once the PE tube was connected to a perfusion device to begin the prograde perfusion of heparin sodium solution (12,500 U of heparin sodium+ 500 ml of NS, 2 ml/min). The pancreatic body tail dissected easily away from the pancreatic head according to the dividing line, and the pancreatic duct and several communicating branches were ligated. Finally, the proximal side of the superior mesenteric vein was carefully ligated, and the portal vein was cut off to harvest the complete pancreatic body tail, which was then stored at − 80 °C for subsequent perfusion decellularization.

## Decellularization procedure

After thawing at room temperature, the isolated pancreatic body tail was connected to a perfusion device to allow for anterograde perfusion at 3 ml/min, and the solutions flowed through the left gastric artery, into the splenic artery, and throughout the vasculature of the pancreas. A 0.02% trypsin (Solarbio)/0.05% EDTA (Sigma-Aldrich) solution (30 min), double-distilled water (15 min), a 0.2 mM PBS solution (15 min), and a mixture of nonionic surfactants (1% Triton-X100 (Solarbio)/0.05% EDTA (Sigma-Aldrich)/0.1% PMSF (Beyotime) were then used as perfusates for rinsing cells and cell debris out of the pancreas. The color of the pancreas changed from white to translucent (approximately 180 min), the subsequent perfusion steps involved an ionic detergent solution (0.1% sodium dodecyl sulfate (SDS) (Sigma-Aldrich) in 0.1 mM PBS) (30 min). A solution of benzonase (90 U/ml, Sigma) was then perfused for 30 min, and this was followed by a final washing step in which 10% fetal bovine serum (FBS, Life Technologies) in PBS with Pen/Strep (100 U/ml) was perfused through the pancreas for an additional 24 h to clear the remaining cellular debris and to sterilize the decellularized scaffolds.

### Evaluation of the native vasculature and circulatory resistance to leaks

The arterial inlet of the decellularized pan-body-tail ECM scaffold was connected to a peristaltic pump (BT100K, Baoding Chong Rui Pump Co., Ltd., China), and diluted methylene blue solution (Y-J Biological) was allowed to flow through the left gastric artery at 2 ml/min and diffuse throughout the vasculature of the scaffold so that the retained native vascular trees could be observed. The absence of blue solution leaking from the pancreatic envelope was considered confirmation of the integrity of the scaffold envelope. This indicated that the circulatory resistance of the scaffold, which is very important for subsequent cell transplantation experiments and a prerequisite for assessing whether a scaffold can survive after in vivo transplantation, was good.

### DNA quantification

These decellularized and natural pancreas were digested with papain solution at 60 °C for 6 h. Papain (Sigma-Aldrich) was dissolved at a concentration of 400 mg/ml in 0.1 M phosphate buffer (pH 6.0) with 5 mM cysteine hydrochloride (Sigma-Aldrich) and 5 mM EDTA (Sigma-Aldrich). This method was employed to produce a lysate for DNA quantification, for hydroxyproline (HxP) assays and to qualitatively measure sGAG. The DNA content was measured via a DNA quantification kit (Quant-iT™ Pico Green® dsDNA Assay kit (Invitrogen)) according to the manufacturer’s instructions. Fluorescence (excitation at 485 nm and emission at 528 nm) was measured on an ELISA reader (Tecan, Männedorf, Switzerland), and the absolute quantity of DNA (ng/ml) was determined against a lambda DNA standard curve (0 to 1000 ng/ml).

### Collagen quantification via hydroxyproline (HxP) assays

A modified HxP assay was used to analyze collagen contents, and the HxP content was estimated to comprise 13.4% of the total collagen content. The lysate contained 80–100 mg of lyophilized tissues and was mixed with 6 M hydrochloric acid (1000 μl) in triplicate. After hydrolysis was performed for 5 h at 100 °C, the mixture was centrifuged, and 50 μl of the hydrolysate was then added to 450 μl of sodium hydroxide citric acid buffer. This solution was further diluted with 4.75 ml of citric acid buffer. Then, 500 μl (in triplicate) of each sample was incubated with 250 μl of chloramine-T reagent, and 250 μl of 6.2 M perchloric acid was added. After the solution was incubated, 250 μl of para-dimethylaminobenzaldehyde was added, and the samples were incubated at 60 °C for 15 min. A standard curve was generated using HxP (Sigma-Aldrich). The absorbances of the samples were measured at a wavelength of 550 nm in a 96-well plate using an ELISA reader (Tecan, Männedorf, Switzerland).

### Characterization of sGAG contents

The sGAG content was measured via a sGAG quantification kit (Blyscan Sulfated Glycosaminoglycan Assay Kit (Biocolor)) according to the manufacturer’s instructions. Briefly, the specimen lysate was mixed with Blyscan dye to bind sGAG. The sGAG-dye complex was then collected via centrifugation. After removing the supernatant and draining the tube, the dissociation reagent was added. Next, 100 μl of each solution was transferred to a 96-well plate. The absorbance against the background control was obtained at a wavelength of 656 nm on an ELISA reader (Tecan, Männedorf, Switzerland), and the amount of sGAG was calculated based on a standard curve obtained using the sGAG standard supplied with the kit.

### Quantitative analysis of cytokines in decellularized pancreases

Total protein was extracted from natural and decellularized pancreases using an ELISA kit (R&D Systems, USA). The concentrations of various cytokines, including EGF, TGF-β, and bFGF, were then measured with an ELISA reader (Tecan, Männedorf, Switzerland) at 450 nm.

### Biocompatibility analysis

All animal experiments were complied with the guidelines of the Institutional Animal Care and Use Committee of Wenzhou Medical University. The biocompatibility analysis described herein was performed following a protocol previously described by Saik-Kia Goh et al. [16]. First, 1-cm^2^ decellularized pancreas constructs were placed within an abdominal subcutaneous pocket in adult Wistar rats at 8–12 weeks of age purchased from the Shanghai Slack Experimental Animal Center. After operation, all animals survived and no complications occurred during the predetermined study period. After 14 days, the rats were anesthetized via inhalation, and the implants along with the surrounding tissues were harvested and fixed in 4% paraformaldehyde for subsequent experiment.

### Cell culture

Rat Aorta PrimaCell™: Normal Aortic Endothelial Cells (NAECs) were purchased from CHI Scientific, Inc., and insulinoma cells (INS-1E) were purchased from ScienCell Research Laboratories. The NAECs were used at passages 5–10. NAECs were cultured in ECM supplemented with 5% FBS, 100 U/ml endothelial cell growth supplement and 100 U/ml penicillin/streptomycin in T75 tissue culture flasks. The INS-1E cells were used at passages 19–26. INS1-E cells were cultured in RPMI 1640 medium supplemented with 10% FBS, 50 μM β-mercaptoethanol and 100 U/ml penicillin/streptomycin in T75 tissue culture flasks. Both cell types were cultured at 37 °C in a 95% air/5% CO_2_ atmosphere.

### Cytotoxicity assay

Decellularized scaffolds were soaked for 24 h in RPMI 1640 medium containing 10% FBS. The culture medium was then collected and used for the culture of INS1-E cells and NAECs. The original culture media alone and with 5% DMSO (Sigma, St. Louis, MO, USA) were employed as controls. After incubation for 24, 48 and 72 h, the cell viability was analyzed via the MTT assay (Sigma). Optical densities (OD) were measured at 570 nm, using 650 nm as a reference wavelength, in a spectrophotometer (Infinite® 200 Pro, Tecan, Austria).

### Recellularization procedure

INS-1E cells (3 × 10^7^) were dissociated and diluted in 3 ml of medium. The cell suspension was then inoculated to the acellular pancreas through the left gastric artery via anterograde perfusion, in three steps of 1 ml each with a 20 min interval between each step. The INS-1E seeded pancreases were subsequently immersed and static cultured in the co-culture medium described above for 2 h before the next step of perfusion culture. To prevent thrombosis, a co-recellularization strategy was adopted using a second cell type, NAECs, before the in vivo transplantation experiment. Similarly, NAECs (3 × 10^7^ cells) were dissociated and diluted in 3 ml of medium. The NAEC suspension was then implanted into the acellular pancreas through the portal vein via retrograde perfusion. The two cell types were seeded sequentially, with same multistep repopulation described for single-cell type seeding. The cells were allowed to attach for 2 h before the introduction of a gentle rinse through the left gastric artery to wash out any unattached cells. The medium was collected after the first perfusion, and the number of single INS-1E cells was counted after centrifugation. The cell-seeded pancreas was placed in a bioreactor as shown in the Fig. 7a. The inlet of the recellularized pancreas was connected to a peristaltic pump with a perfusion rate of 1 ml/min. The remaining part of the recellularized pancreas was left immersed in a co-culture medium. The medium in the perfusion system was changed every other day. The seeded pancreases were cultured in the perfusion system under static conditions at 37 °C with a 95% air/5% CO_2_ atmosphere for 4 days.

### Glucose-stimulated insulin secretion assay

The insulin-secreting function of seeded INS-1E cells was tested by GSIS [20]. The control group was an attachment culture group that was cultured in the original culture medium, and the perfusion culture group was cultured in a perfusion system as described above. The cells were stimulated with 2 mM and 20 mM glucose to provoke insulin secretion. Insulin accumulation in the medium was measured after 30 min using a rat insulin ELISA kit (Enzyme-linked Biotechnology Co., Ltd., China). The amount of secreted insulin was normalized against total protein.

### In vivo transplantation

A diabetic model was established in adult Wistar rats via intraperitoneal injection of streptozotocin, as described by Goyal SN et al. [21], with a high and stable blood glucose level. Internal jugular vein catheterization was performed on the recipients preoperatively. The diabetic rats were divided into three groups (*n* = 18): group I (*n* = 6) consisted of diabetic rats without interference, group II (*n* = 6) consisted of rats that underwent transplantation of a recellularized pancreas (30 × 10^6^ cells) with connected vessels, and group III (*n* = 6) consisted of rats that underwent implantation of INSL-1E cells (30 × 10^6^ cells) via the portal vein. The diabetic recipients were anesthetized via ether inhalation, and sterile conditions were maintained during the operations. A laparotomy was performed (Additional file 2), and heparinization was first accomplished through the injection of heparin (250 U/ml, 0.2–0.4 ml/100 g) into the inferior vena cava. Nephrectomy on the left kidney was performed with an empty kidney region, and the left renal artery and vein were retained as the grafted vessels. The recellularized pancreas was placed within the kidney region, and the arterial inlet was connected to a peristaltic pump to perfuse heparin sodium solution (12,500 U of heparin sodium+ 500 ml of NS) for several minutes before in vivo transplantation. The outlet of the recellularized scaffold was then connected to the recipient’s renal vein to perfuse the heparin sodium solution for several minutes to avoid thrombosis. The recipient’s renal vein and the inlet of the recellularized scaffold were blocked with vascular clips. The inlet of the recellularized scaffold was subsequently connected to the recipient’s renal artery. Finally, the vascular clips were removed, and whether the blood flow in the recellularized scaffold was unobstructed was observed. The incision was closed after confirmation that there was no bleeding around the transplanted pancreas. Hypocoagulability was maintained through internal jugular vein injection of heparin (250 U/ml, 0.2–0.4 ml/100 g) for 3 days postoperatively. After surgery, fasting glucose levels were measured every 6 hours on the first day and then every day using a glucometer (Model C158981 l653, Security type, three Connaught Biology Sensing Co., Ltd., China).

### Histological examination

Natural pancreas, decellularized pancreas matrix, and recellularized pancreas samples collected at day 4 and transplanted pancreas samples collected at day 1 and day 7 were fixed with 4% paraformaldehyde, embedded in paraffin, and processed for hematoxylin and eosin staining.

### Immunohistochemistry procedures

Tissue samples of natural pancreas and decellularized pancreas matrixes were fixed with 4% paraformaldehyde, embedded in paraffin, and cut into 7-mm-thick sections.

Endogenous peroxidase activity in the rehydrated sections was quenched with 3% hydrogen peroxide, and this step was followed by blocking with 5% normal goat serum for 1 h (ZLI-9021, ZSGB-BIO). The primary antibodies were diluted in antibody diluent (ZLI-9028, ZSGB-BIO) and incubated on the slides overnight at 4 °C. The primary antibodies included anti-laminin (ab11575, Abcam), anti-collagen I (ab34710, Abcam), anti-collagen IV (ab6586, Abcam), anti-fibronectin (ab45688, Abcam), anti-insulin (ab181547, Abcam), and anti-Von Willebrand factor (ab11713, Abcam) antibodies. The negative controls lacked a primary antibody. The secondary antibodies employed were anti-rabbit IgG (PV-6001, ZSGB-BIO) and anti-mouse IgG (PV-6002, ZSGB-BIO). After diaminobenzidine chromogen (ZLI-9017, ZSGB-BIO) was developed, the sections were washed with distilled water and mounted with Histomount (ZLI-9550, ZSGB-BIO).

### Immunofluorescence staining of the recellularized pancreas

Decellularized and recellularized pancreas samples were prepared for immunofluorescence analysis following standard protocols as described previously. The tissue samples were fixed with 4% formaldehyde (Thermo Fisher), protected with 15% sucrose overnight followed by 30% sucrose for 12 h, and then cut into 10-μm-thick sections. For immunostaining, the primary antibodies used as described above. The secondary antibodies employed were donkey anti-rabbit Alexafluor 488 (1:500, Thermo Fisher) and donkey anti-goat Alexafluor 594 (1:500, Thermo Fisher). After staining with the first primary antibody overnight at 4 °C, the slides were washed three times with 0.1 M PBS (5–10 min) the slides before incubating with the second primary antibody. The slides were washed again three time before mounting with the ProLong® Gold Anti-Fade Reagent with DAPI (Invitrogen). Represent Images were captured with an Olympus BX51 fluorescent microscopy (Olympus Corporation, Tokyo, Japan).

### Scanning electron microscopy (SEM) and transmission electron microscopy (TEM)

Natural, decellularized and recellularized pancreases were fixed in 2.5% glutaraldehyde in 0.1 M PBS (pH 7.4) for 60 min. The samples were then thoroughly washed three times with 0.1 M PBS for 15 min each, fixed in 1% osmium tetroxide (OsO4) in 0.1 M PBS for 60 min, and washed three times with PBS for 15 min each. The samples were subsequently dehydrated in a gradient series of alcohol with 15 min for each step. The samples were then critical point dried and coated with Au/Pd using a Cressington Coater 108A sputter coater. Electron microscopy images were obtained using a emission SEM. For TEM, the tissue samples were fixed in 2.5% glutaraldehyde in PBS. The tissue samples were post-fixed in 1% OsO_4_ in PBS, dehydrated through a graded series of acetone and embedded in Epon. Thin (60-nm) sections were cut, mounted on 200-mesh copper grids and counterstained with 2% aqueous uranyl acetate for 7 min and 1% aqueous lead citrate for 2 min. The samples were finally observed with a transmission electron microscope.

### Statistical analysis

All experiments were performed with at least three biological replicates, with all assays performed in triplicate. Quantitative data are expressed as the means ± SD. Significant difference among groups were determined with the Wilcoxon rank-sum test for two-group comparisons or ANOVA followed by post hoc analysis for multiple-group comparisons by using SPSS 16.0 software (IBM, USA). Probability value of *P* < 0.05 was considered to be statistically significant.

## Results

### Harvest and decellularization of the pancreatic body tail

**Fig. 1:**
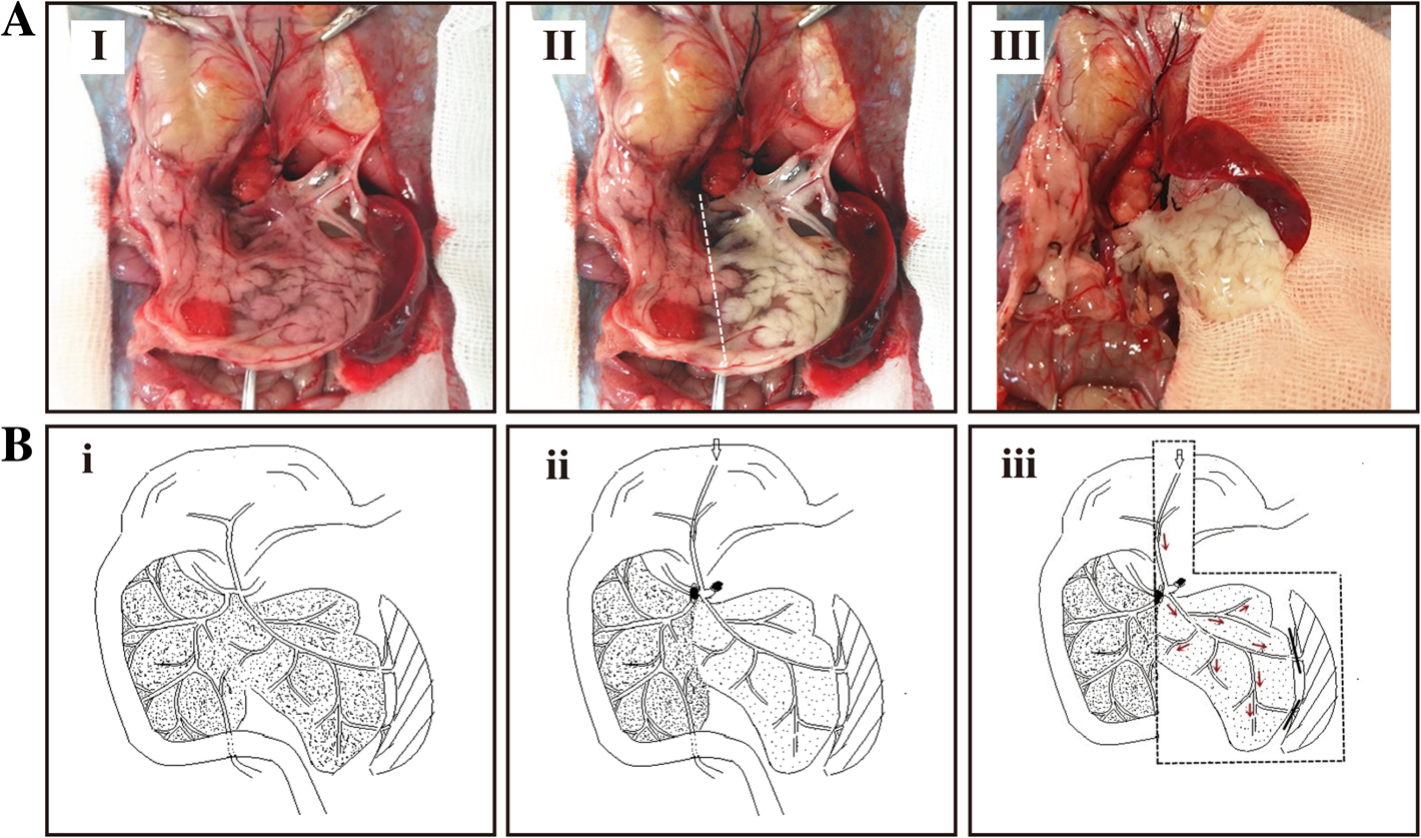
Method used to harvest rat pancreatic body tails: **a** (I-III) After a left gastric artery catheter was successfully inserted, celiac and hepatic arteries were ligated, forming a significant boundary between the head portion and the body-tail of the pancreas. A peristaltic pump was connected to pump saline into the target portion of the pancreas according to the boundaries established for harvesting the pancreatic body tail. **b** (i-iii) Schematic of the method used to ligate the celiac and common hepatic arteries after the left gastric artery was successfully catheterized and saline was pumped from the left gastric artery

**Fig. 2:**
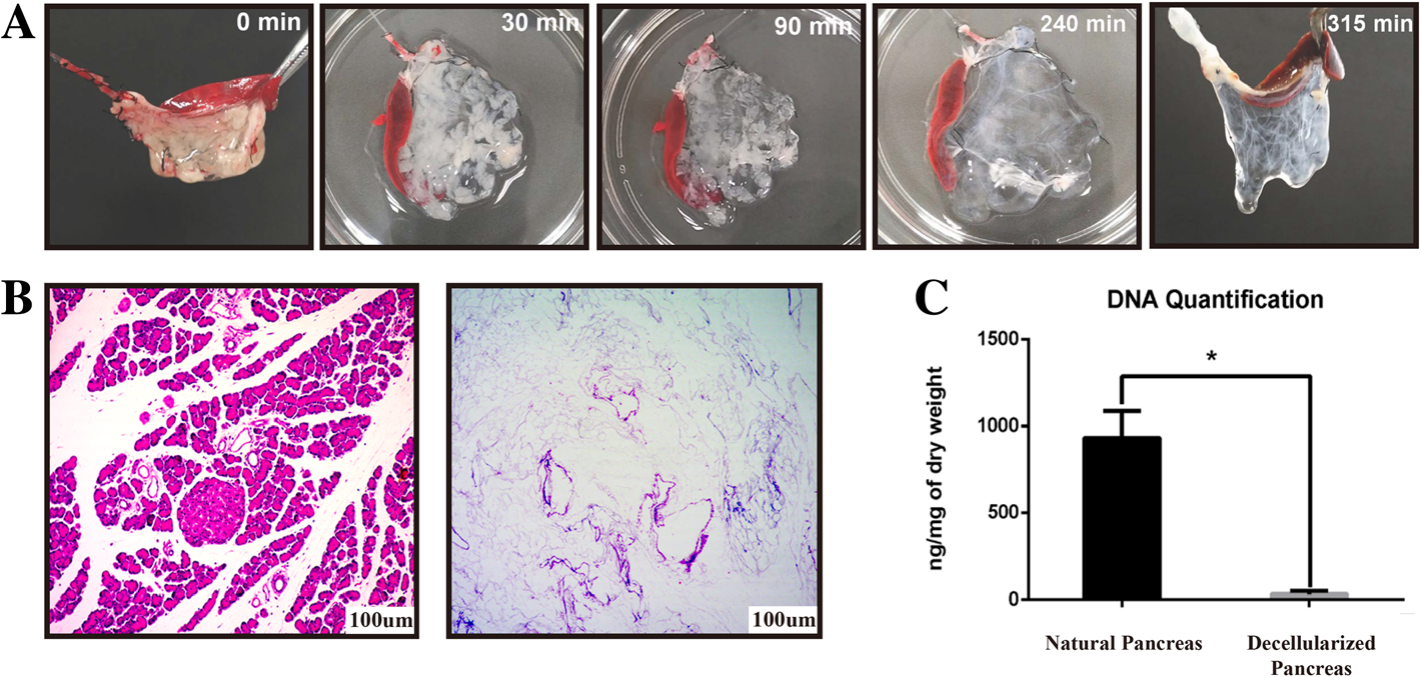
Decellularization steps: (**a**) Decellularization of the pancreatic body tail scaffold. Images illustrate the gradual change in color caused by perfusion-decellularization in mouse pancreases. Treatment with 1% triton x-100 resulted in a decellularized pancreas after 315 min. **b** H&E staining showing that there were no remnant cells after the completion of decellularization. **c** DNA quantification demonstrating that the amount of DNA was clearly lower in the treated organs than in natural organs (from 931.9 ± 267.8 to 11.7 ± 3.6 ng/mg), *P* < 0.05

As shown in Fig. 1a, after the No. 10 PE tube was successfully inserted into the left gastric artery, the pancreatic body tail became white when the celiac artery and the common hepatic artery were ligated during perfusion with heparin sodium solution. The harvesting of the pancreatic body tail was easily achieved according to the boundary between the pancreas head and body tail. Under the perfusion strategy applied in our study, the partial pancreas displayed a gradual change in color from normal pink to mostly white and finally became completely translucent, and this process took a total of 315 min (Fig. 2a). H&E staining showed no remnant cells after the completion of decellularization (Fig. 2b). DNA quantification demonstrated an obvious reduction of DNA compared with that of the natural organ (from 931.9 ± 267.8 to 11.7 ± 3.6 ng/mg, *P* < 0.001), and the percent of DNA residues in the scaffold was less than 3% of natural organ. Fig. 2c shows direct evidence of successful decellularization.

### Characterization of vasculature and circulatory resistance to leaking

**Fig. 3:**
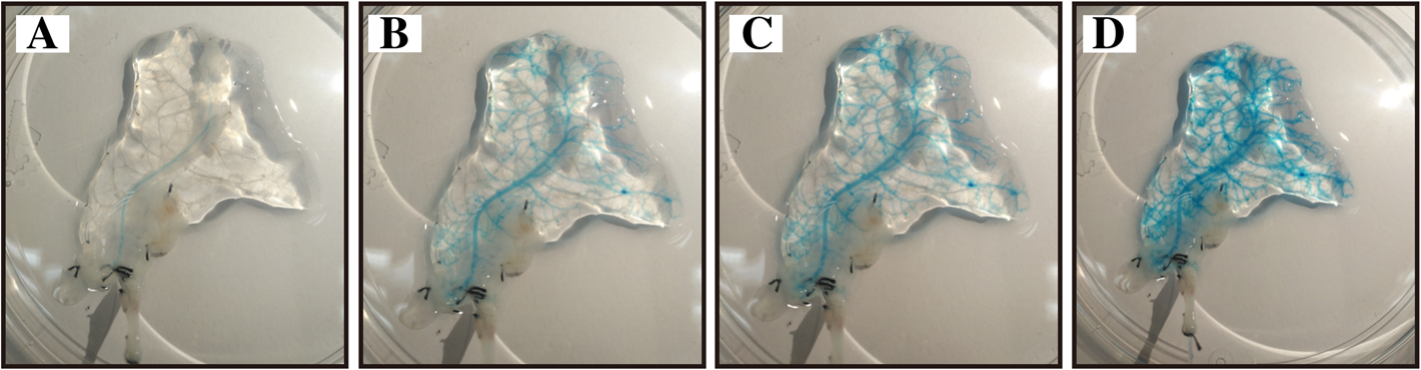
Evaluation of the native vasculature and circulatory resistance to leaks: (**a**-**d**) Diluted methylene blue solution was allowed to flow through the left gastric artery and to diffuse throughout the vasculature of the scaffold so that the retained native vascular trees could be evaluated. No blue solution leaked out of the envelope, confirming the integrity of the scaffold envelope

The integrity of the vasculature within the acellular scaffold was verified via anterograde perfusion of methylene blue dye through the left gastric artery. We observed rapid diffusion from the arterial inlet to the venous outlet and throughout the entire clear vascular tree and no leakage from the pancreatic envelope (Fig. 3a-d).

### ECM composition of the pancreatic acellular scaffold

**Fig. 4:**
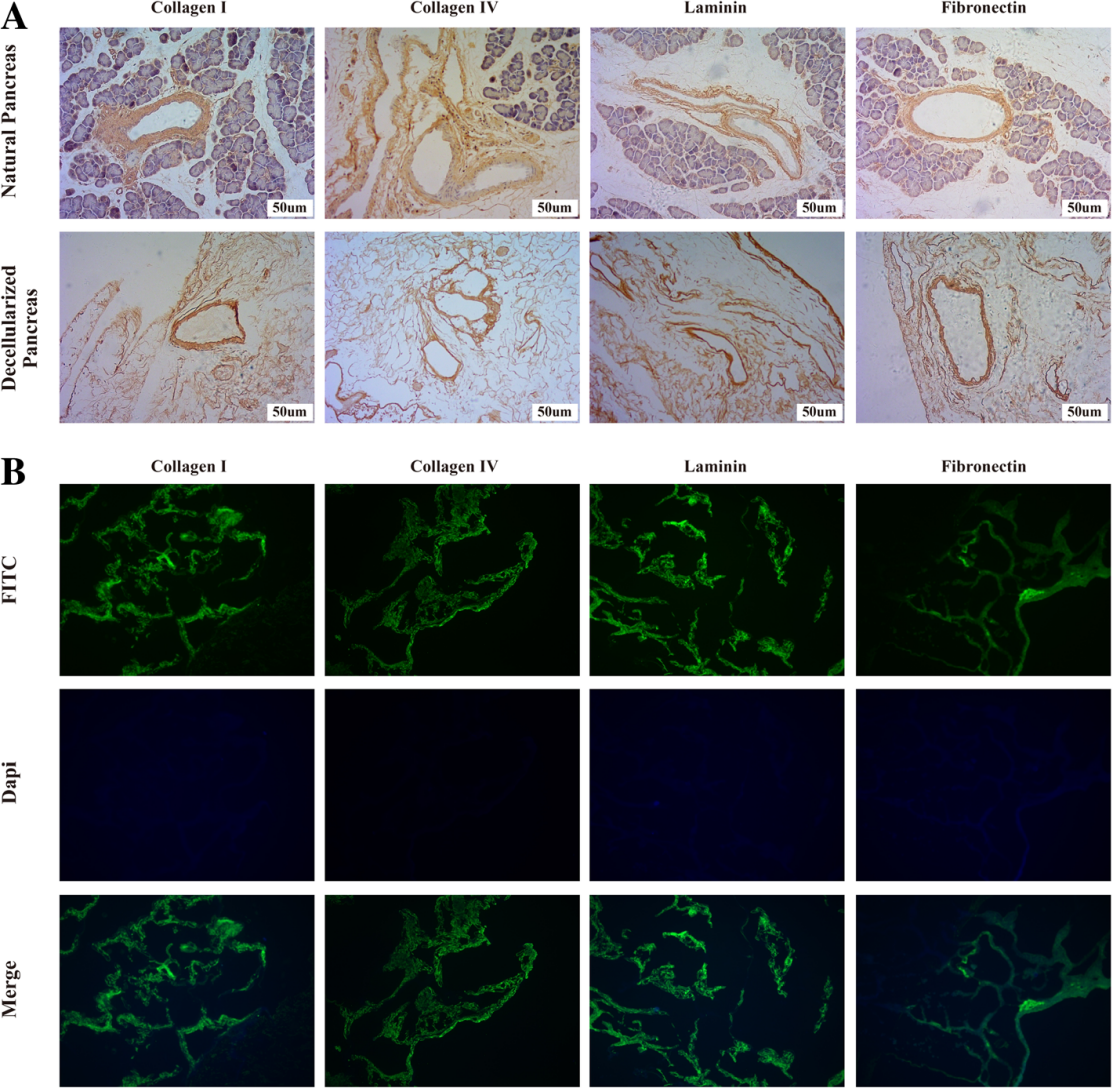
ECM composition of the pancreatic acellular scaffold: (**a**-**b**) Immunohistochemical and immunofluorescence staining of ECM components showing that four main collagens were retained after the decellularization process. These included collagen I, collagen IV, fibronectin and laminin

The natural tissue microenvironment composed of complete tissue-specific ECM in the acellular pancreas is regarded as a major advantage for the survival and proliferation of seeded cells. Immunohistochemical and immunofluorescence staining of ECM components showed that our acellular scaffold preserved four main collagens, including collagen I, collagen IV, fibronectin and laminin, after the decellularization process, and these were widely distributed in the basement membrane or around the pancreatic large and small vascular and ductal structures (Fig. 4a&b). This finding suggested that our decellularization method could completely preserve the ECM composition in a similar manner.

**Fig. 5:**
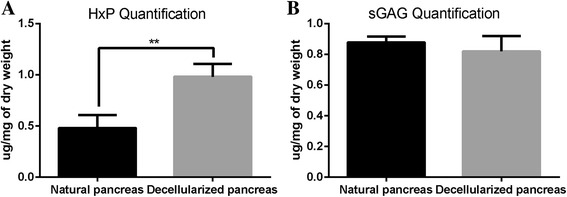
Quantitative determination of ECM composition: (**a**) the hydroxyproline content was significantly higher in the treated pancreatic tissue (0.98 ± 0.13 μg/mg) than in fresh pancreatic tissue (0.48 ± 0.13 μg/mg) (P < 0.05). **b** The retention of s sGAG in the decellularized pancreas (0.878 ± 0.37) was not significantly different from that observed in natural pancreases (0.819 ± 0.1) (*P* > 0.05)

Additionally, quantitative determination of the ECM composition was performed. The total collagen content was measured using a modified HxP assay, and the results showed that the content of hydroxyproline was significantly increased (0.98 ± 0.13 μg/mg) compared with that in fresh pancreatic tissue (0.48 ± 0.13 μg/mg). This difference was statistically significant (*P* < 0.05) (Fig. 5a). In addition, a Blyscan assay was used to assess quantitatively the sGAG content, and the retention of the sGAG in the decellularized pancreas (0.878 ± 0.37) showed no significant difference from the natural pancreas (0.819 ± 0.1) (*P* > 0.05) (Fig. 5b). The higher amounts of collagen and sGAG in the decellularized pancreas compared with the natural pancreas were justifiable considering that the cellular components were removed from the scaffolds.

### Biophysical and chemical properties: Spatial structure, growth factors and tissue and cell compatibility

**Fig. 6:**
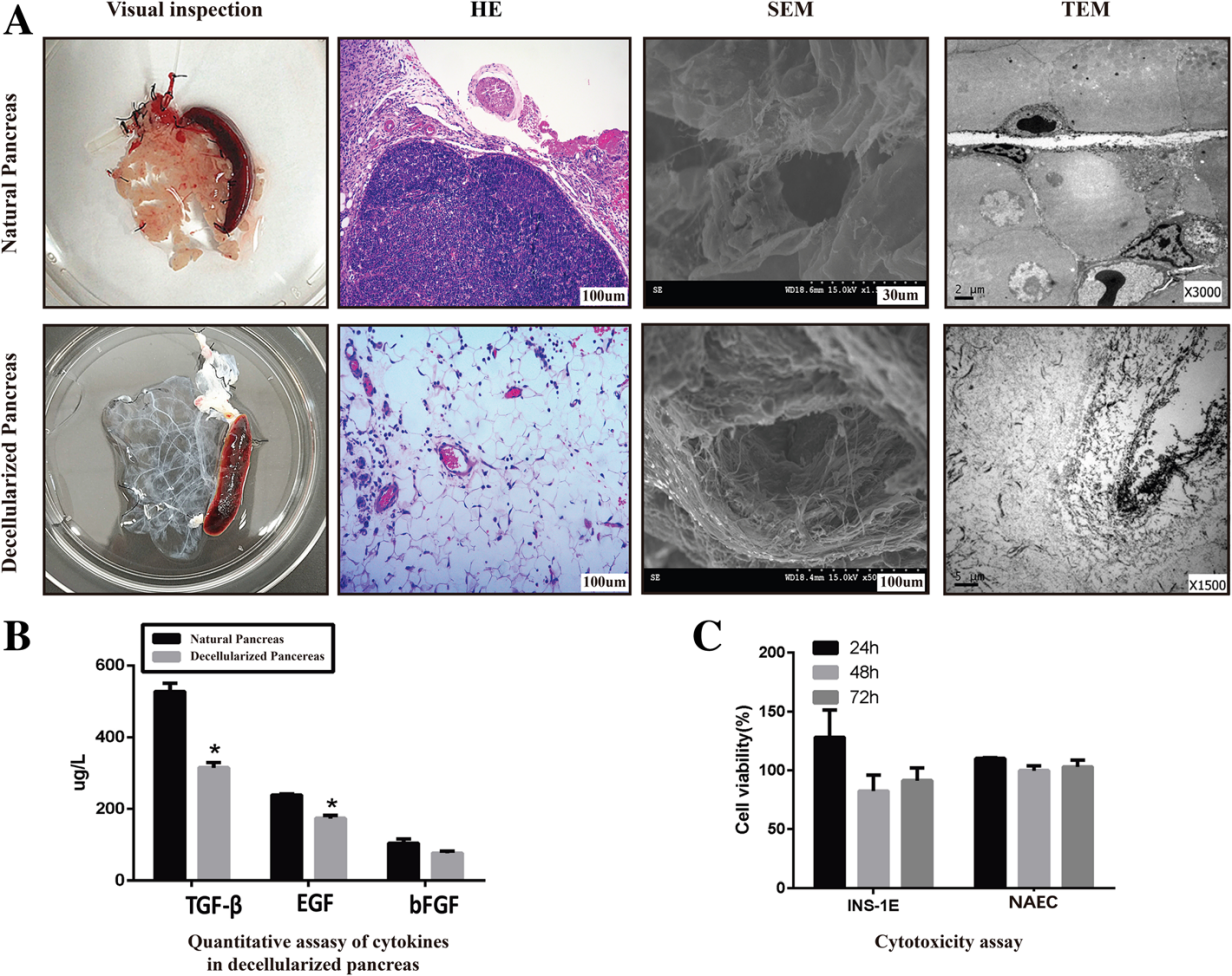
Biophysical and chemical properties: (**a**) The gross appearance of a natural pancreas and a decellularized pancreas. H&E staining showed that there were no pathological signs of an immune rejection response in the transplanted acellular scaffold. SEM and TEM results showed that micro-vascular structures were retained after decellularization. **b** ELISA results suggested that most of the growth factors were preserved in decellularized pancreases. **c** MTT tests showed that the acellular scaffold is non-toxic to cells and suitable for co-cultured cells

Scanning electron microscopy (SEM) revealed the 3D spatial structure and microenvironment of the pancreatic acellular scaffold after the decellularization procedure. Transmission electron microscopy (TEM) depicted the intact micro-vascular structure of the acellular pancreas and showed the interstitial space with organized fibers of collagen (Fig. 6a).

A quantitative analysis via ELISA showed that several growth factors (EGF, TGF-β, and bFGF) were preserved to a considerable extent in the scaffold after decellularization (Fig. 6b). These molecules are well-established essential factors in cell development, neuronal growth, regeneration, angiogenesis and vasculogenesis [13].

In the subcutaneous implantation experiment, a histological analysis 2 weeks post-surgery showed that the acellular scaffold was compatible with the host, similar to other efficient acellular ECM scaffolds [22, 23], and no multinucleate foreign body giant cells or other pathological signs of an immune rejection response were detected (Fig. 6a).

The MTT test showed that 24 h after inoculation, the acellular scaffold leaching solution did not significantly inhibit the growth of co-cultured cells but rather promoted cell proliferation. A possible explanation for this finding might be that the growth factors were retained within the scaffold. After 48 and 72 h, growth decreased slightly. However, there were generally no significant differences between the three time points (Fig. 6c). These results suggested that the acellular scaffold exhibited good compatibility with and a Non-cytotoxic effect on co-cultured cells.

### Recellularization of the pan-body-tail ECM scaffolds

#### INS-1E islet cells

**Fig. 7:**
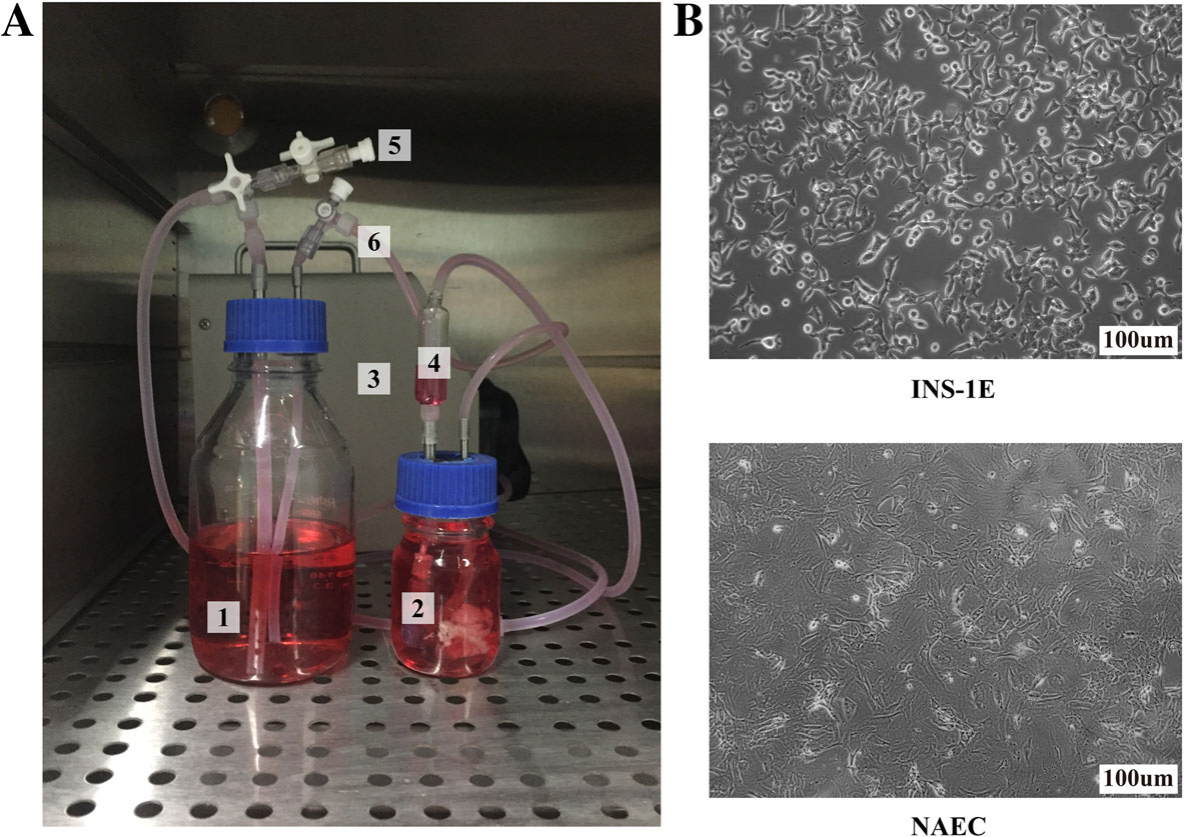
Recellularization steps: (**a**) Perfusion culture system: 1-Reservoirs, 2-Perfusion chamber, 3-Peristalic pump, 4-Bubble trapper, 5-Inlet valve, and 6-outlet valve. **b** Cells used for recellularization

**Fig. 8:**
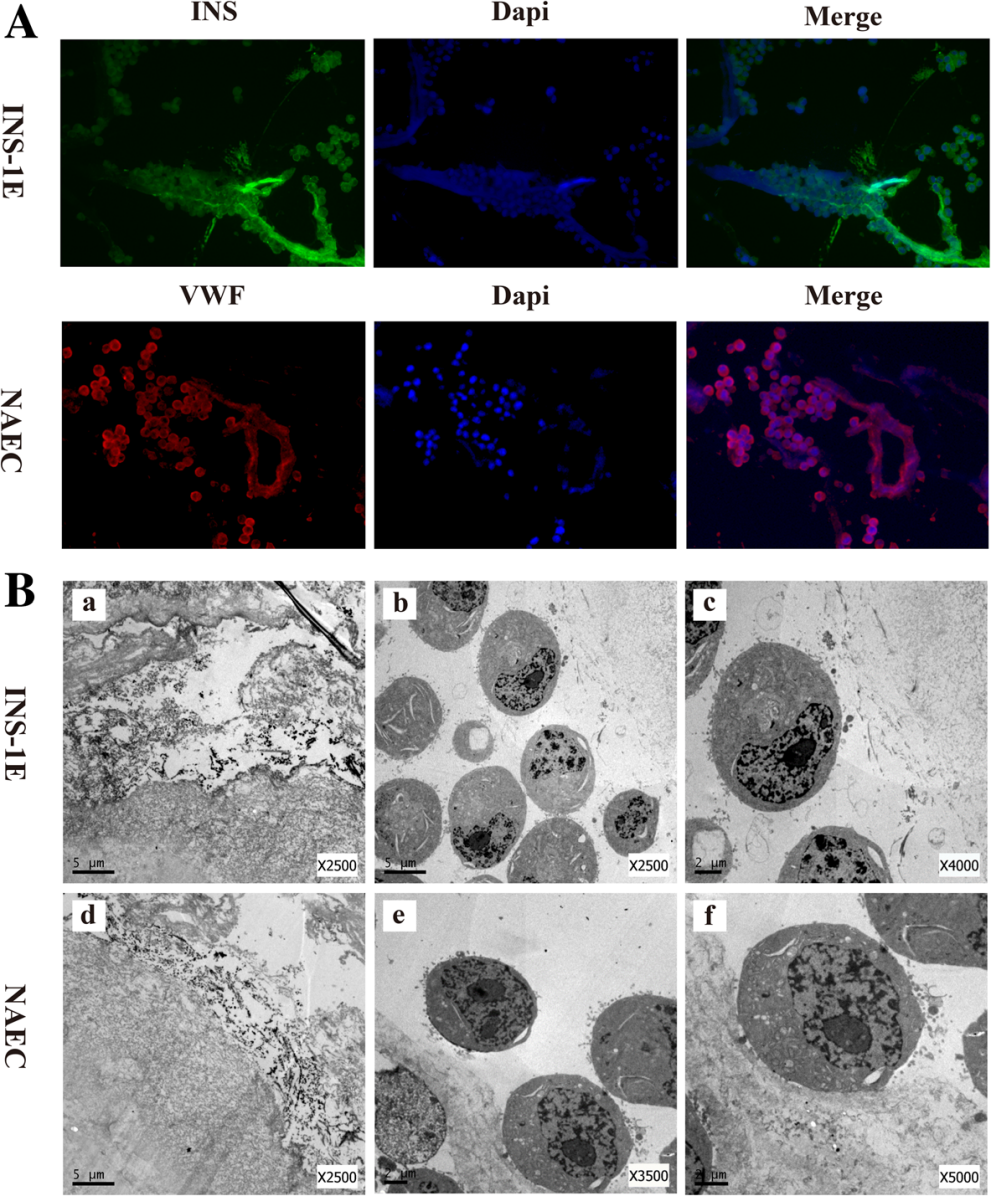
Verification of Recellularization: (**A**) Immunofluorescence staining of a recellularized pancreas. The recellularized rat pancreas was stained with DAPI nuclear staining (blue), INS for INS-1E cells (green) and vWF for NAEC cells (red). **B** TEM in a recellularized pancreas showing that the INS-1E cells tended to aggregate into groups and that the NAEC cells were prone to colonize and adhere tightly within the parenchymal space

**Fig. 9:**
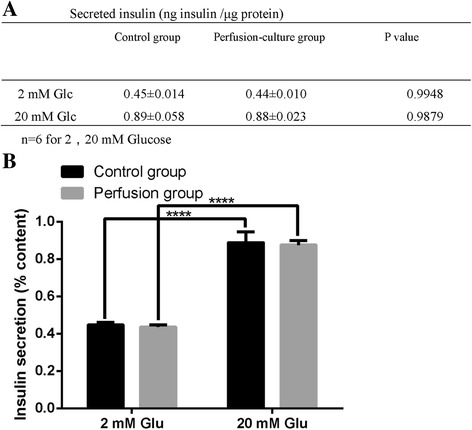
Glucose-stimulated insulin secretion assay: (**a**) There was no significant difference in the amount of secreted insulin at basal conditions (2 mM glucose) or after stimulation with 20 mM glucose between the control and perfusion-culture groups. **b** Insulin secretion is presented as a % content, and the results showed that insulin levels were higher in animals stimulated with 20 mM glucose than at basal conditions (2 mM glucose) in both the control group and the perfusion-culture group. The error bars show the SEMs, *n* = 6. ****, *P* < 0.01. The amount of secreted insulin was normalized against total protein levels

Two hours after the three-step injection of INS-1E cells (30 × 10^6^) into the pan-body-tail ECM scaffold, the small number of cells that were not captured by the scaffold were counted ((0.73 ± 0.49) × 10^6^ cells, *n* = 3), and the result indicated an approximately 97% efficiency of deposition of the INS-1E cells within the scaffold. During cell seeding, an obvious color change was observed, particularly from the main vasculature to its branches, indicating orderly cell delivery. The recellularized pancreas was analyzed for engraftment and survival after 4 days of perfusion culture in the perfusion culture system as the picture shows (Fig. 7a). Immunofluorescence staining showed that INS-1E cells tended to localize around the vessels (Fig. 8A). TEM showed that INS-1E cells tend to aggregate into groups (Fig. 8B). The GSIS results showed no significant differences in the amounts of insulin secretion among the attachment culture group and the perfusion culture group, demonstrating the cell compatibility of the scaffolds (Fig. 9a&b).

#### NAEC endothelial cells

The pan-body-tail ECM scaffold was seeded with NAECs (30 × 10^6^) using a method similar to that described above before in vivo transplantation. TEM showed that the cells were apparently colonized and adhered tightly within the parenchymal space. This finding demonstrates the endothelial nature of the seeded cells, as evidenced by vWF staining (Fig. 8A&B).

### In vivo transplantation

After left renal resection was completed, the recellularized pancreas construct could be transplanted to the region of the left kidney, and the cannulations of the arterial inlet and venous outlet were successfully connected with the renal artery and vein, respectively. When the vascular tree inside the graft was well perfused and no bleeding was detectable, the abdomen was closed layer by layer. Six rats underwent this transplant surgery, and the average operation time was approximately 90 min, without any deaths during surgery due to graft bleeding. However, one rat manifested sudden severe limb convulsions and died of cardiopulmonary function attenuation 4 hours after the surgery, possibly due to a pulmonary embolism according to the postmortem, which revealed that extensive thrombosis was present in the vascular tree.

### Results of histological examinations

**Fig. 10:**
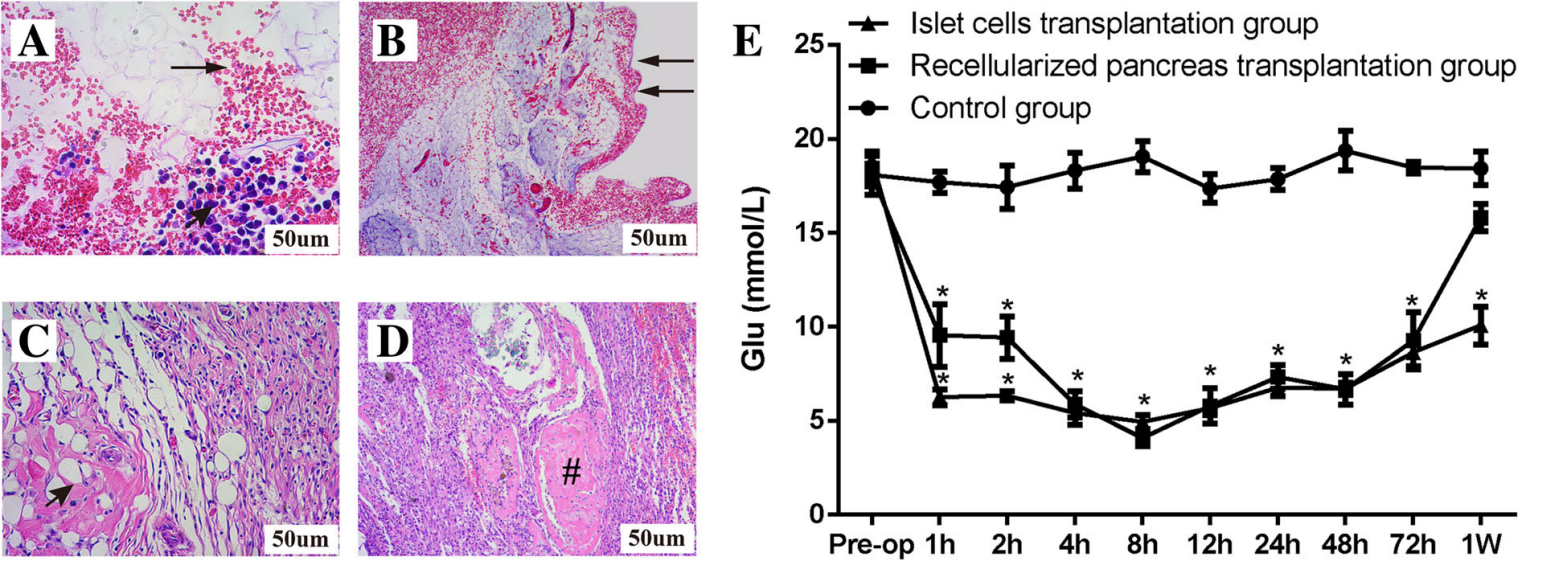
In vivo transplantation: (**a**) H&E staining of transplanted scaffolds showing that the transplanted scaffolds contained a large number of normal red blood cells (arrow) and numerous islet cells (INS-1E) (arrow head). **b** The integrity of the pancreatic capsule (arrow) was completely retained. **c** H&E staining of the scaffolds before transplantation showing a honeycomb structure in the scaffold (arrow). **d** H&E staining of the transplanted scaffolds after 1 week showing thrombosis (#) in the scaffolds. **e** Blood glucose monitoring results showed that blood glucose levels were clearly lower after the transplantation of the recellularized pancreas or the direct injection of islet cells via the portal vein. The transplanted recellularized pancreas maintained normal blood glucose levels for 7 days

One day after transplantation, we observed the histopathology of the grafts. H&E staining showed that the transplanted scaffolds contained a large number of normal red blood cells and numerous islet cells (INS-1E), and that the integrity of the pancreatic capsule was completely retained (Fig. 10a&b). This demonstrated that blood circulation was successfully established between the recipient rats and the grafts and that there were no leaks in the vascular tree. After 1 week, H&E staining showed that the honeycomb structure (Fig. 10c) had disappeared in some areas and that a mass of mononuclear cells surrounded the partially degraded ECM, which showed large red dye-stained areas, possibly indicating thrombosis (Fig. 10d).

### Blood glucose monitoring after transplantation in diabetic rats

After surgery, fasting glucose levels were measured in the rats in each group at a specific time point using a glucometer. In group II, fasting glucose levels declined to 9.08 ± 2.4 mmol/l within 2 h of the operation, and 8 h later, they had decreased to 4.7 ± 1.8 mmol/l (*P* < 0.05). Blood glucose levels fluctuated and gradually increased to 11.3 ± 4.8 mmol/l at 1 week after the operation (Fig. 10e). The results of blood glucose monitoring confirmed that the recellularized pancreases of the rats in group II, which were transplanted with the vessels connected, survived and played a role in controlling blood sugar levels by establishing a pathway for insulin secretion, even though the duration they maintained normal blood glucose levels was not particularly long.

## Discussion

Pancreas transplantation, which was first performed by Kelly et al. [24], has been shown to improve the secondary complications of diabetes compared with insulin treatment [25, 26] and quality of life [27]. In addition, it yields higher rates of insulin independence than islet transplantation [28]. Despite these advantages, donor shortage, surgical morbidity and the need for life-long immunosuppression significantly limit clinical applications. In recent years, with the advent of decellularization technology, pancreatic tissue engineering and regenerative medicine have developed rapidly, to the extent that the construction of a transplantable bioengineered endocrine pancreas based largely on an ideal pancreatic acellular ECM scaffold has become the main goal of the present research in this field [29–32]. Herein, we describe a novel pancreatic ECM scaffold generated from the rat pancreatic body tail, which is much easier to harvest and more efficient to decellularize than the whole pancreas. We demonstrated that this natural pancreas ECM scaffold preserved not only the complete ECM elements and most biochemical properties but also the intact 3D spatial architecture. Our data were consistent with the results of a mouse study by Saik-Kia Goh [15], and the novel pancreatic ECM scaffold retains a closed vascular network, including an independent arterial inlet and venous outlet and can be used for in vivo transplantation. Thus, this scaffold shows its potential as a platform for pancreatic tissue engineering.

Many recently developed methods, including the use of chemical reagents, enzymology and osmotic pressure gradient perfusion, have improved the decellularization procedure [33, 34]. In our study, we chose freeze-thawing and trypsin hydrolysis as necessary steps because these two steps can distinctly enhance perfusion efficiency according to other studies [35–37]. The main chemical agents used for this purpose are SDS and Triton X-100. SDS is an ionic detergent that is more effective for thicker organs such as kidneys and hearts, whereas the deionized detergent Triton X-100 is more efficient for thin tissues, such as blood vessels and skin [23]. A study addressing the preparation of the rat liver acellular scaffold showed that using SDS as the detergent was less efficient and more time-consuming, resulting in increased destruction of the ECM composition and a greater than 50% loss of aminoglycans [38]. In contrast, the milder nonionic detergent TritonX-100 not only achieves a decellularized effect similar to that of SDS but also reduces the damage to ECM collagens [39]. Therefore, in the present study, based on the fact that natural pancreas tissue is loose and soft, we optimized the combination of perfusion detergents and found that using TritonX-100 as the main detergent (180 min) supplemented with SDS for a short time (30 min) can decrease the total damage to the scaffold from the detergents. In addition, we selected anterograde perfusion through the left gastric artery, in line with the original direction of the physiological flow and the corresponding fluid shear stress inside the vessel. Furthermore, compared with portal vein or pancreatic duct, perfusion through the left gastric artery allowed harvesting of the pancreatic body tail as the main body for decellularization due to the clear physiological boundary between the pancreatic head and body tail after ligating the common hepatic artery and celiac trunk artery during perfusion (Fig. 1a&b). Additionally, the volume of the pancreatic body tail is significantly smaller than that of the whole pancreas, which could reduce the diffusion distance required for perfusates to reach the cells and promote removal of cells and cell debrisfrom the tissue [40, 41]. Consequently, we greatly decreased the perfusion flow rate to reduce the perfusion pressure, which could further reduce the fluid shear stress responsible for vascular matrix damage and might also protect the internal vascular network and the external pancreatic envelope of the scaffold. These modified techniques allowed efficient generation of a novel acellular scaffold, which required approximately 315 min at a flow rate of 3 ml/min.

The results of our experiments using the above strategy for decellularization showed that there was no cell debris residue in the pancreatic ECM scaffold, as demonstrated by H&E staining and SEM. First, quantitative DNA analysis showed that less than 50 ng of DNA was preserved per mg of ECM dry weight, indicating successful decellularization according to previously defined stringent requirements [23]. These requirements are crucial because residual DNA fragments in the ECM scaffold have been demonstrated to cause adverse immunological responses upon transplantation in vivo [42, 43]. Second, our results showed that using an improved method of decellularization achieved the purpose of maximally preserving biologically active ECM components. IHC staining showed that our decellularization protocol preserved most essential structural proteins. Furthermore, SEM analysis showed that most ECM proteins retained their physiological organization after decellularization, and this allowed the maintenance of the native 3D microenvironment. These environments have been shown to be specific to particular anatomical locations, where they support site-specific cell attachment and functions [44–48]. Third, the cytotoxicity and biocompatibility of the ECM scaffold must be taken into consideration when using it for in vitro culture and in vivo transplantation. The results shown here demonstrate that the ECM scaffolds were non-toxic and biocompatible. Finally, several studies have shown that the ECM also contains many growth factors (GFs) that promote cell adhesion and tissue structural integration, repair, and proliferation [10, 49–54]. An evaluation of the biological activity of the scaffolds showed that the decellularized scaffolds retained some GFs, including EGF, bFGF and TGF-β that are necessary for angiogenesis and for promoting cell maturation, neuronal growth and regenerative immune mediation. Additionally, according to the results of MTT tests, most of the GFs were bioactive. Several previous studies have achieved successful repopulation using different types of pancreatic acellular scaffolds, including positive results in studies using seeded islet cells [13–15]. In the present study, the pan-body-tail ECM scaffold was easily recellularized with INS-1E cells by perfusing cultures through the preserved vascular network in the above-described bioreactor. We demonstrate that INS-1E cells were effectively seeded into the pancreatic ECM and that they maintained the ability to secrete insulin. Blood glucose monitoring results also revealed that the seeded INS-1E cells survived and functioned after the recellularized pancreas was transplanted.

The most innovative characteristic of our pan-body-tail ECM scaffold, in contrast to the presently available whole pancreatic acellular scaffolds, is the functional integrity of its vascular network, which has an independent arterial inlet and a venous outlet from the left gastric artery to the portal vein. Additionally, the overall circulation of the scaffold is leak-proof, and this is another important consideration when using pancreatic decellularization to develop feasible in vivo transplantation applications. In the present study, methylene blue perfusion staining initially demonstrated that the vascular network inside the engineered scaffold was clear and intact. To the best of our knowledge, no previous study in the bioengineering literature has reported the transplantation of a recellularized pancreas based on a whole decellularized pancreas scaffold. There are two obstacles to implementing this strategy. (1) First, it is difficult to use a whole pancreatic acellular scaffold for in vivo transplantation because its vessels must be connected to those of the recipient and the anatomy of the pancreatic vasculature is complicated, particularly at the pancreas head; and (2) second, of all parts of the scaffold, the pancreas head is the most likely to leak because it is difficult to isolate it from the duodenum because of the complicated and extensive communicating branches between them. However, in our pan-body-tail ECM scaffold, the pancreas head was discarded, and the remaining scaffold could be used on its own, thereby avoiding these obstacles. In the present study, we successfully transplanted recellularized pan-body-tail ECM scaffolds into recipient diabetic rats. To prevent thrombosis formation inside the grafts after transplantation, we performed co-recellularization of endothelial cells (NAECs) and perfusion heparinization of the scaffold before transplantation. We combined this strategy with treating the recipient with intermittent anticoagulation therapy (heparin sodium) several days after transplantation. The data showed that the blood glucose levels of the diabetic rats were significantly lower and that they maintained a euglycemic state for nearly 1 week. These data indicated that effective blood circulation had developed between the recellularized scaffolds and the vasculature of the recipient diabetic rats and that the INS-1E cells could be stimulated to secrete insulin in response to high glucose levels in the blood, thus playing a hypoglycemic role. The results of H&E staining showed that at 24 h after transplantation, the grafts still contained normal erythrocytes, and the INS-1E cells were surrounded by an intact pancreas envelope, demonstrating that functional blood circulation had been established with the recipient rats and that there was no leakage from the grafts. After 1 week, H&E staining showed that the honeycomb structure had disappeared, leaving only red dye-stained areas maybe thrombus which possible cause of the implant failure only after 1 week. The recellularized artificial endocrine pancreas described here is far from an ideal donor tissue for persistent therapy because INS-1E cells are not the optimal choice for in vivo transplantation and heparinization cannot be employed for long-term anticoagulation therapy. However, our results demonstrate that it is feasible to using our novel scaffold for in vivo transplantation, and further efforts should be directed towards the construction of an ideal artificial endocrine pancreas based on this pan-body-tail ECM scaffold. Future studies should place an emphasis on the islet cell source and vascular endothelialization.

## Conclusions

The current study describes a novel pancreatic ECM scaffold prepared from the rat pancreatic body tail via perfusion through the left gastric artery. Thorough characterization revealed that the resulting pancreatic ECM scaffold described herein maximally protected the ECM composition and maintained an intact vascular tree and reasonable resistance to leakage, showing good biocompatibility, bioactivity and support of seeded islet cells. We further showed the pioneering possibility of in vivo circulation-connected transplantation of a recellularized pancreas based on this novel scaffold, although only in the short term. To the best of our knowledge, no research group has yet been able to generate a functional bioengineered endocrine pancreas that can be successfully used for in vivo transplantation. Therefore, by providing such a promising pancreatic ECM scaffold, the present study might represent a key improvement and have a positive impact on endocrine pancreas bioengineering.

## Additional files

Additional file 1: **Figure S1.** (A) Anatomical structure of the rat pancreas. (B) Schematic diagram of surgical procedures for rat pancreatectomy. (TIFF 2979 kb)

Additional file 2: **Figure S2.** In vivo transplantation steps: (A-C) Procedure of recellularized pancreas transplantation. Firstly heparinization was accomplished through the injection of heparin into the inferior vena cava. Nephrectomy on the left kidney was performed with an empty kidney region, and the left renal artery and vein were retained as the grafted vessels. The recellularized pancreas was placed within the kidney region, and the arterial inlet was connected to a peristaltic pump to perfuse heparin sodium solution for several minutes before in vivo transplantation. The outlet of the recellularized scaffold was then connected to the recipient’s renal vein to perfuse the heparin sodium solution for several minutes to avoid thrombosis. The recipient’s renal vein and the inlet of the recellularized scaffold were blocked with vascular clips. The inlet of the recellularized scaffold was subsequently connected to the recipient’s renal artery. Finally, the vascular clips were removed, and whether the blood flow in the recellularized scaffold was unobstructed was observed. The incision was closed after confirmation that there was no bleeding around the transplanted pancreas. (TIFF 8673 kb)

## Abbreviations

bFGF: Basic fibroblast growth gactor
DAPI: 4′,6-diamidino-2-phenylindole
DMSO: Dimethyl sulfoxide
ECM: Extracellar matrix
EDTA: Ethylenediaminetetraacetic acid
EGF: Epidermal growth factor
Elisa: Enzyme linked immunosorbent assay
FBS: Fetal bovine serum
GFs: Grow factors
Glc: Glucose
GSIS: Glucose-stimulated_insulin_secretion
H&E: Hematoxylin-eosin staining
HxP: Hydroxyproline
IHC: I Mmunohistochemistry
INS: Insulin
INS-1E: Insulinoma cells
MTT: 3-(4,5)-dimethylthiahiazo (−z-y1)-3,5-di- phenytetrazoliumromide
NAEC: Normal aortic endothelial cells
NS: Normal saline
OD: Optical densities
Pan-body-tail: Pancreatic body tail
PBS: Phosphate-buffered saline
PE: Polyethylene
Pen/Strep: Penicillin streptomycin
PMSF: Phenylmethanesulfonyl fluoride
SD: Sprague Dawley
SDS: Sodium dodecyl sulfate
SEM: Scanning electron microscopy
sGAG: Sulfate glycosaminoglycan
TEM: Transmission electron microscopy
TGF-β: Transforming growth factor-β
vWF: von Willebrand factor
