# Supplementary figures and images for: The rat pancreatic body tail as a source of a novel extracellular matrix scaffold for endocrine pancreas bioengineering
Source: J Biol Eng. 2018 Apr 27;12:6. doi: 10.1186/s13036-018-0096-5 (PMC5923185; doi:10.1186/s13036-018-0096-5)

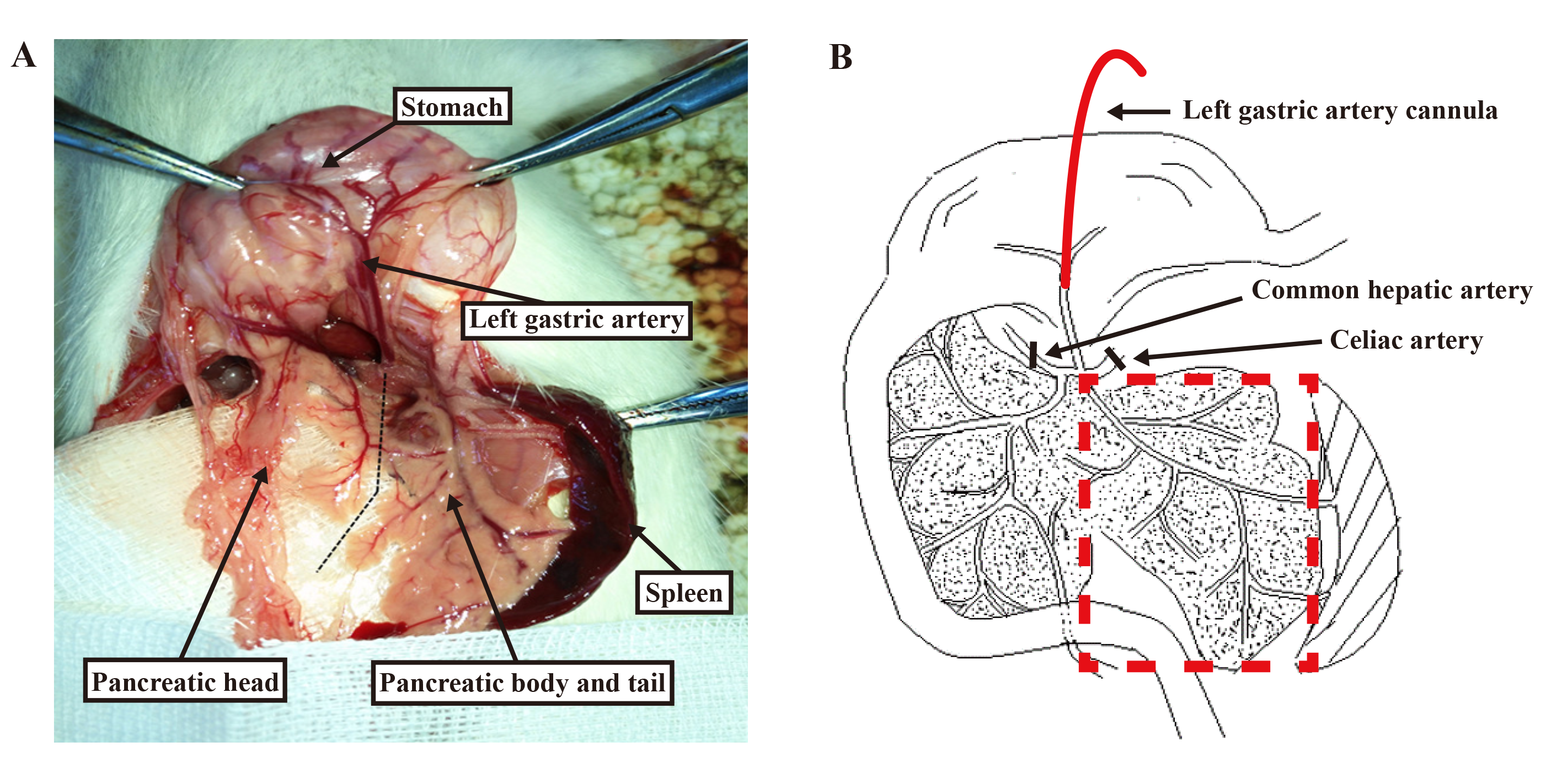

Supplement: Supplementary file 1 — Figure S1. (A) Anatomical structure of the rat pancreas. (B) Schematic diagram of surgical procedures for rat pancreatectomy. (TIFF 2979 kb) [file 13036_2018_96_MOESM1_ESM.tif]

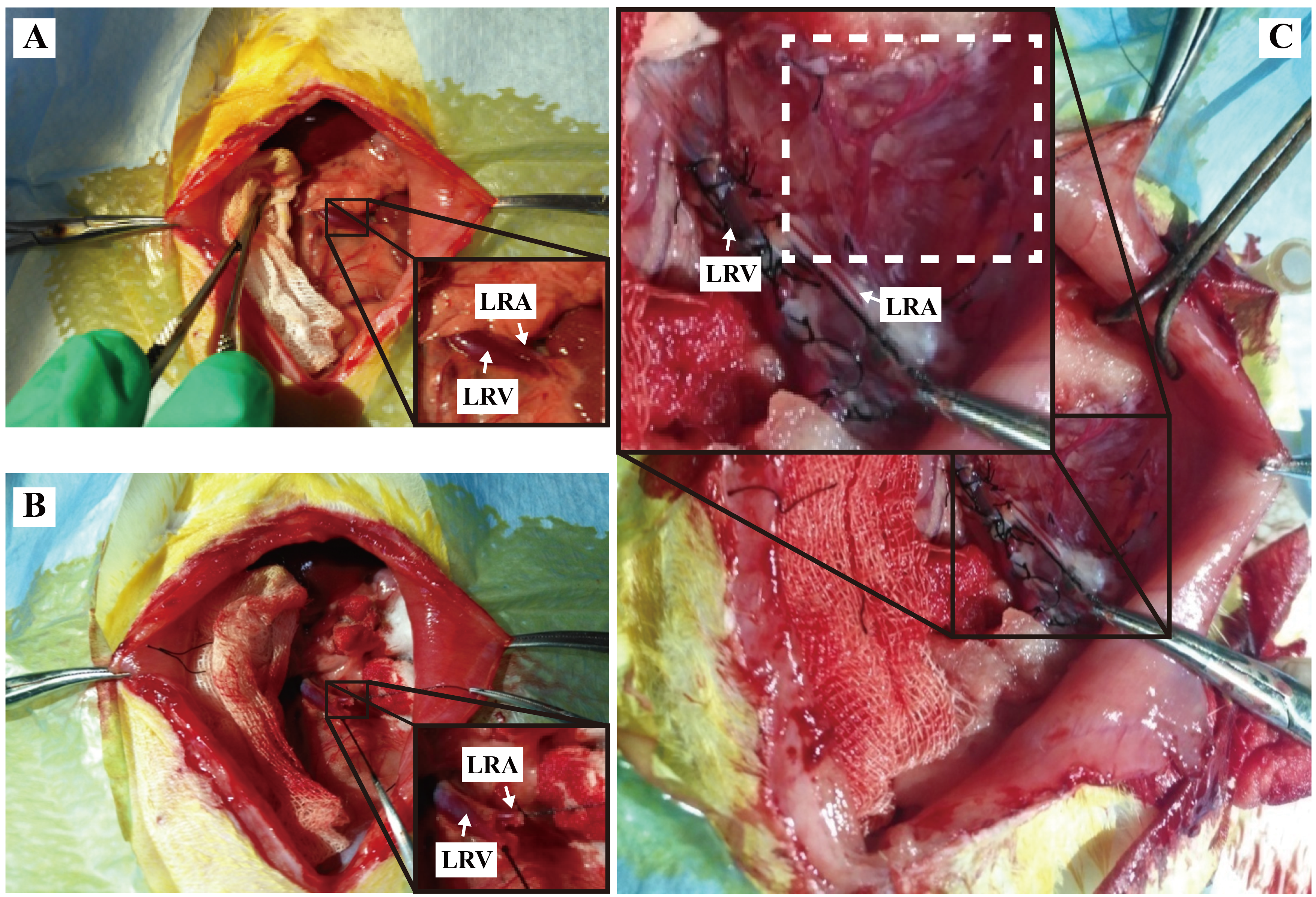

Supplement: Supplementary file 2 — Figure S2. In vivo transplantation steps: (A-C) Procedure of recellularized pancreas transplantation. Firstly heparinization was accomplished through the injection of heparin into the inferior vena cava. Nephrectomy on the left kidney was performed with an empty kidney region, and the left renal artery and vein were retained as the grafted vessels. The recellularized pancreas was placed within the kidney region, and the arterial inlet was connected to a peristaltic pump to perfuse heparin sodium solution for several minutes before in vivo transplantation. The outlet of the recellularized scaffold was then connected to the recipient’s renal vein to perfuse the heparin sodium solution for several minutes to avoid thrombosis. The recipient’s renal vein and the inlet of the recellularized scaffold were blocked with vascular clips. The inlet of the recellularized scaffold was subsequently connected to the recipient’s renal artery. Finally, the vascular clips were removed, and whether the blood flow in the recellularized scaffold was unobstructed was observed. The incision was closed after confirmation that there was no bleeding around the transplanted pancreas. (TIFF 8673 kb) [file 13036_2018_96_MOESM2_ESM.tif]
